# Si/PEDOT:PSS Hybrid Solar Cells with Advanced Antireflection and Back Surface Field Designs

**DOI:** 10.1186/s11671-016-1560-0

**Published:** 2016-08-08

**Authors:** Yiling Sun, Zhenhai Yang, Pingqi Gao, Jian He, Xi Yang, Jiang Sheng, Sudong Wu, Yong Xiang, Jichun Ye

**Affiliations:** 1 School of Energy Science and Engineering, University of Electronic Science and Technology of China, Chengdu, 611731 People’s Republic of China; 2 Ningbo Institute of Materials Technology and Engineering, Chinese Academy of Sciences, 1219# Zhongguanxi Road, Zhenhai District, Ningbo, Zhejiang Province 315201 People’s Republic of China

**Keywords:** Si/PEDOT:PSS, Hybrid solar cells, Simulation, Antireflection layer

## Abstract

Molybdenum oxide (MoO_3_) is one of most suitable antireflection (AR) layers for silicon/poly(3,4-ethylenedioxythiophene):poly(styrenesulfonate) (Si/PEDOT:PSS) hybrid solar cells due to its well-matched refractive index (2.1). A simulation model was employed to predict the optical characteristics of Si/PEDOT:PSS hybrid solar cells with the MoO_3_ layers as antireflection coatings (ARCs), as well as to analyze the loss in current density. By adding an optimum thickness of a 34-nm-thick ARC of MoO_3_ on the front side and an effective rear back surface field (BSF) of phosphorus-diffused *N*^+^ layer at the rear side, the hybrid cells displayed higher light response in the visible and near infrared regions, boosting a short-circuit current density (*J*_sc_) up to 28.7 mA/cm^2^. The average power conversion efficiency (PCE) of the Si/PEDOT:PSS hybrid solar cells was thus increased up to 11.90 %, greater than the value of 9.23 % for the reference devices.

## Background

Organic/inorganic hybrid solar cells that combine the advantages of crystalline silicon (c-Si) and organic solar cells have attracted much attention in recent years owing to their solution-based treatment, simplified processing, and routinely increased power conversion efficiency (PCE) [1–3]. The key role of the organic materials in a hybrid solar cell is to form a heterojunction with Si [4, 5]. Among numerous materials, PEDOT:PSS, with its electron blocking and hole transporting characteristics, seems to be a promising candidate for organic *p*-type layer [6, 7]. Moreover, PEDOT:PSS is amenable to solution-based processes and shows good conductivities of up to 1000 S/cm, suitable work functions in the range of 4.8–5.2 eV, and high transparency in a visible light range [8–11]. All of these advantages enable silicon/poly(3,4-ethylenedioxythiophene):poly(styrenesulfonate) (Si/PEDOT:PSS) hybrid solar cells to achieve a PCE above 13 % [12–17], up to now. Aiming to improve the performance of the Si/PEDOT:PSS hybrid solar cells to the theoretical boundary [18], a range of research activities have been carried out, e.g., antireflection (AR) layer coating [19–23], PEDOT:PSS property tuning [24, 25], Si surface texturing [26–28], and back surface field (BSF) layer forming [18, 29].

Adding an AR layer seems to be an effective way to improve the PCE of Si/PEDOT:PSS hybrid cells, with the mechanism of taking advantage of the difference in optical path between the top and bottom sides of the AR layer. As a result, the reflection was reduced and the transmission was enhanced. Ultimately, higher intensity of the light reaching the absorber results in higher short-circuit current density (*J*_sc_). However, limited by the quite lower reflective index (1.2–1.6) for the PEDOT:PSS layer, it is critical to precisely figure out appropriate materials and optimum thickness of the AR layer. Meanwhile, special designs are also needed at the rear side of the hybrid solar cells in order to efficiently collect the photo-generated carriers related to the incident light with a longer wavelength.

In this study, we selected a molybdenum oxide (MoO_3_) film as an antireflection (AR) layer because of its suitable refractive index (~2.1) for the Si/PEDOT:PSS device. By simulating the light absorption and transmission behavior of the MoO_3_ AR layer of different thicknesses in different wavelength ranges, we obtained a more precise thickness value without the hassle of intensive experimenting. For the purpose of forming a uniform film and without damaging the interface on the PEDOT:PSS film, we deposited the MoO_3_ layer by thermal evaporation. Besides, a phosphorus-diffused BSF layer was applied onto a medium-doped substrate to suppress recombination and to promote the carrier collection efficiency at the rear side. Furthermore, gendering by the simulated results, we reached an optimum thickness of 34 nm experimentally. The Si/PEDOT:PSS solar cells with BSF and the MoO_3_ AR layer we fabricated exhibited better light response and external quantum efficiency (EQE) within the wavelength range of 500–1100 nm, resulting in obviously improved performance in comparison with the reference cells, with the *V*_oc_ from 572.6 to 599.8 mV, the *J*_sc_ from 23.3 to 28.7 mA/cm^2^, and the PCE from 9.23 to 11.90 % under the simulated solar illumination (AM 1.5 G, 100 mW/cm^2^).

## Methods

In this study, we predicted the optical performance of solar cells utilizing the full-wave finite-element method which solves Maxwell’s equations within a unit cell surrounded by periodic boundary condition and perfectly matched layers [30]. The wavelength-dependent refractive index (*n*) of a Si material was taken from Palik’s data [31], and the spectral response in the wavelength range of 300–1200 nm for hybrid solar cells was considered. The overall performance was evaluated with standard AM 1.5 G illumination under normal incidence. The photocurrent density (*J*_ph_), total current density (*J*_tot_), total loss percentage (*P*_loss_), loss current density (*J*_loss_), reflection loss (*R*), and parasitic absorption loss were calculated by integrating the absorption and reflection spectrum of the solar cell [15, 32].

**Fig. 1:**
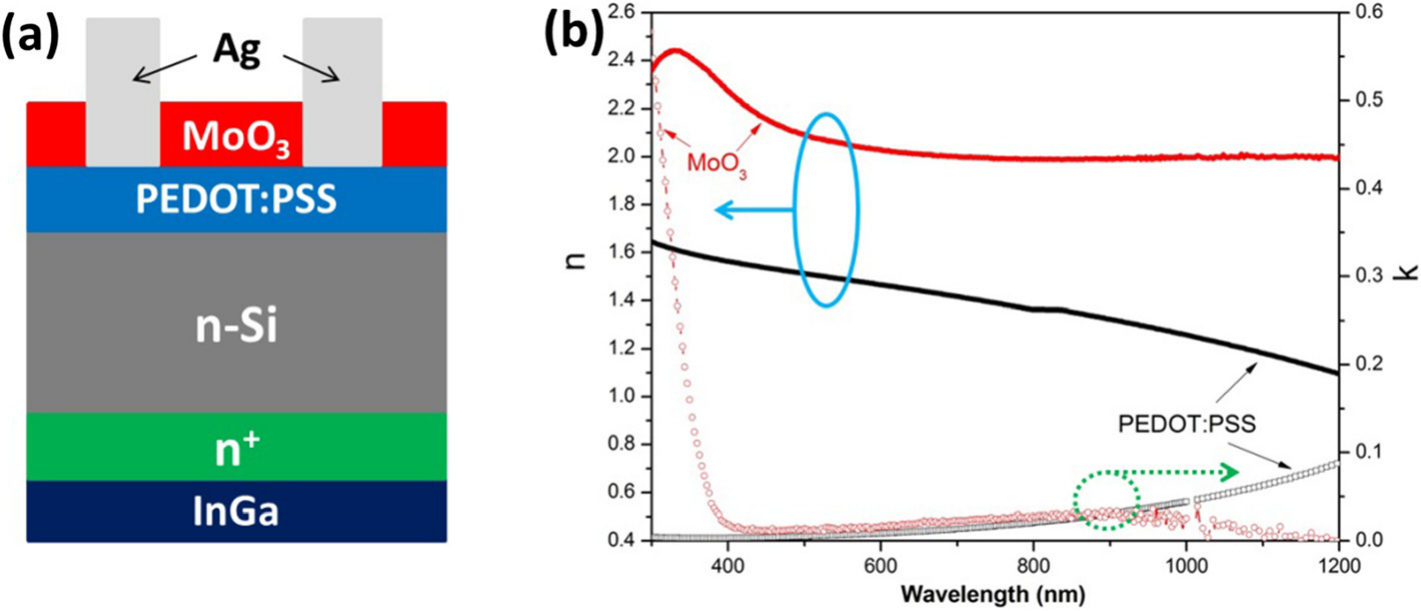
**a** The schematic diagram of a Si/PEDOT:PSS hybrid solar cell. **b** The *n* and *k* value curves of the PEDOT:PSS and thermal-evaporated MoO_3_ AR layers

The cross-sectional structure of the cells is schematically shown in Fig. 1a. First, the *n*-type (100) Si-substrate (single side polished, float zone, 20 × 20 mm, thickness 300 ± 15 μm, resistance 1–5 Ω cm) was cleaned using RCA1, RCA2 [33], and 8 % (volume ratio) HF cleaning procedures. The BSF was then formed by phosphorus diffusion from a POCl_3_ source in a quartz-tube furnace at 850 °C on both sides. After diffusion, the phosphor-silicate glass and front doped region were removed by HF and HF HNO_3_ mixed solution, respectively. Next, the PEDOT:PSS solution was spin-coated on the front side of the wafer at the speed of 5500 rpm for 60 s. The samples were then heated on a hotplate at 125 °C for 15 min to remove the solvents. After that, a 150-nm-thick silver grid electrode was deposited on the surface of the PEDOT:PSS layer via thermal evaporation, while an InGa eutectic layer was used as the rear electrode to provide ohmic contact. Finally, the thermal-evaporated MoO_3_ AR layer was deposited on the top of the device.

The thickness of the spin-coated PEDOT:PSS film was obtained by a step profiler (Veeco Dektak150). The refractive index and extinction coefficient (*k*) of thermal-evaporated MoO_3_ AR layer were tested with spectroscopic ellipsometry (J.A. Woollam M-2000 DI). Using a spectrophotometer (Helios LAB-re, with an integrating sphere), the reflectivity of the PEDOT:PSS and MoO_3_ layers was measured in the wavelength range of 400–1100 nm. After the irradiation intensity was calibrated using a standard silicon photovoltaic device (Oriel, model 91150V), the current density-voltage (*J-V*) characteristic of the hybrid solar cells was tested with a Keithley 2400 digital source meter (Keithley) under simulated sunlight (100 mW/cm^2^) illumination provided by a xenon lamp (Oriel) with an AM 1.5 filter. The open area of the cells was 0.7 cm × 0.8 cm, with 0.11 cm^2^ shaded by the grid of Ag electrodes. The Newport silicon detector and 300 W Xenon Light Source with a spot size of 1 × 3 mm were used to measure the EQE.

## Results and Discussion

**Fig. 2:**
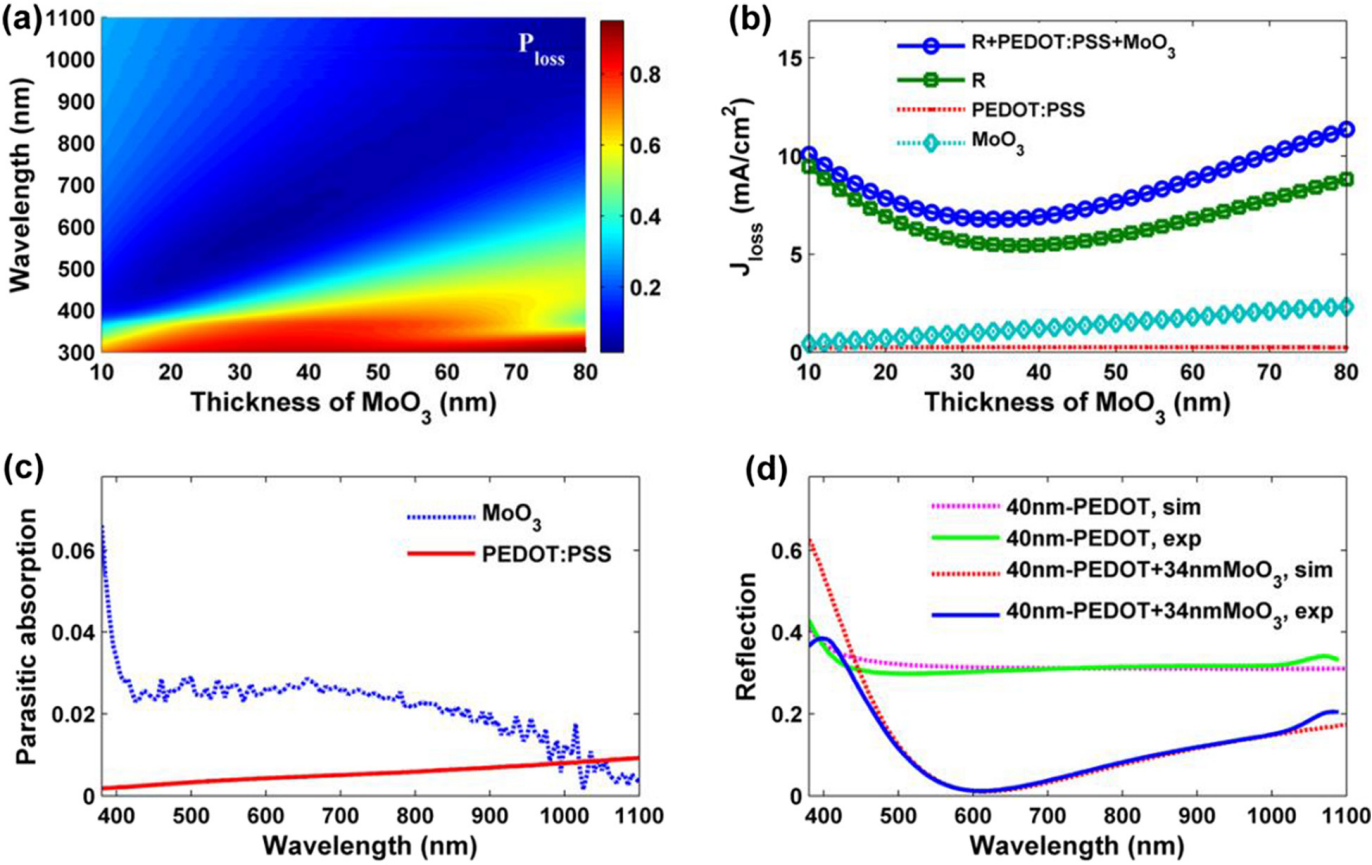
**a** Simulated loss percentage mapping of total current density with a stack of MoO_3_/PEDOT:PSS/Si-substrate. The thickness for the PEDOT:PSS layer is fixed at 40 nm and the thicknesses for MoO_3_ layer is tunable ranging from 10 to 80 nm. **b** Simulated loss current density curves of total loss current density (R + PEDOT:PSS + MoO_3_), the reflection loss current density(*R*), the parasitic absorption loss caused by PEDOT:PSS and MoO_3_ with fixed thickness of 40 nm for the PEDOT:PSS layer, and tunable thicknesses ranging from 10 to 80 nm for the MoO_3_ layer. **c** Simulated parasitic absorption curves of MoO_3_ and PEDOT:PSS at wavelength range of 350–1100 nm. **d** Experimental and simulated reflectance spectra of PEDOT:PSS/Si-substrate, MoO_3_/PEDOT:PSS/Si-substrate

The thickness of the PEDOT:PSS film on the polished wafer was tested as about 40 nm under the condition of 5500 rpm for 60 s. The *n* and *k* value curves of the PEDOT:PSS thermal-evaporated MoO_3_ AR layer tested by spectroscopic ellipsometry are shown in Fig. 1b. Combining an earlier simulation report [30, 34] and total current density (*J*_tot_) value of 43.77 mA/cm^2^ which is obtained by integrating the sun’s spectrum from 300 to 1100 nm, we mapped the loss percentage of total current density percentage (*P*_loss_) using different colors (as shown in Fig. 2a). This figure depicts the light response from both the PEDOT:PSS and the MoO_3_ layers, with fixed thickness (40 nm) for PEDOT:PSS and tunable thicknesses for the MoO_3_ layer. It was observed that *P*_loss_ is relatively large in the short wavelength. In most visible and infrared ranges, the film displayed lower and almost constant reflectivity with the increase in thickness; specifically, the light *P*_loss_ was below 0.1 in this range for the majority of the thickness range.

To further investigate the optimum thickness of the MoO_3_ AR layer, we simulated the *J*_loss_-thickness curve quantitatively (Fig. 2b). And we also refined the loss current; it can be divided into three parts, namely the reflection loss current density (*R*), parasitic absorption loss caused by PEDOT:PSS, and MoO_3_. As the thickness of PEDOT:PSS is constant, its parasitic absorption is almost fixed. Compared with reflection loss, the materials’ parasitic absorption loss are rather small with the increasing of MoO_3_ thickness. Such small parasitic absorptions also can be predicted by the *k* curves in Fig. 1b. In general, the tendency of the total current loss is consistent with reflection loss which results in the optimal thickness of approximately 34 nm. Considering the *J*_tot_ value of 43.77 mA/cm^2^, the *J*_ph_ of the hybrid cell is 37.00 mA/cm^2^.

The parasitic absorptions of PEDOT:PSS and MoO_3_ are in the wavelength range of 350–1100 nm, as illustrated in Fig. 2c. It can be found that MoO_3_ displays higher absorption especially in the ultraviolet band. Both MoO_3_ and PEDOT:PSS have small parasitic absorptions. It corresponds to the *k* curve in Fig. 1b and also supports the small current loss caused by parasitic absorptions in Fig. 2b.

To match the simulated and experimented refractive indices, we simulated the reflectivity of PEDOT:PSS heterojunction and with a 34-nm-thick MoO_3_ layer (Fig. 2d). It can be found that the simulated curves fitted well with the experimental ones in the wavelength range of 400–1100 nm. Both the simulated and experimented data showed much lower refractive indices after the deposition of 34 nm MoO_3_ compared with the single PEDOT:PSS layer. Combining the small parasitic absorptions of PEDOT:PSS and MoO_3_, the great reduction of reflectivity in full-wave band will result in the enhancement of short-circuit current obviously.

**Fig. 3:**
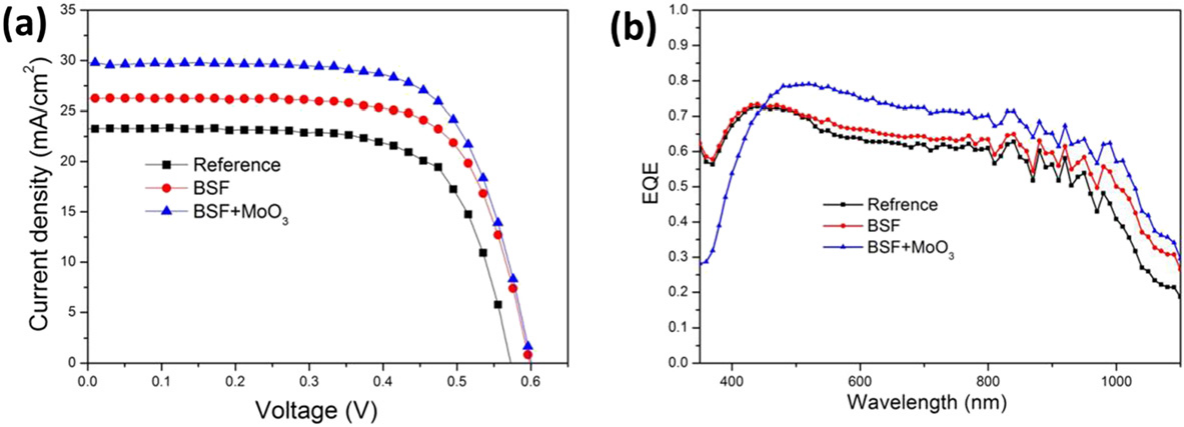
**a** Current density versus voltage characteristic of the hybrid solar cells from reference, BSF cell, and BSF and MoO_3_ cells under 100-mW/cm^2^ illumination (AM 1.5 G). **b** EQE curve of Si/PEDOT:PSS cells with reference, BSF layer, and BSF and MoO_3_ layers

Figure 3a depicts the light *J-V* curves of three types of cells, namely the reference sample, the sample with a BSF layer, and the sample with a front MoO_3_ AR layer and a rear BSF layer. Five cells were fabricated for each type, and their electrical performance was measured. The average values of *J*_sc_, *V*_oc_, fill factor (FF), and PCE of the fabricated devices are summarized in Table 1. The introduction of a highly doped *N*^+^ layer between the *n*-Si and back-side metal electrode delivers a BSF that effectively suppresses the recombination rate at the rear surface via a downward band bending for reflecting holes. The inducing of the BSF layer is aiming for the better use of the photon-generated carrier. Therefore, apart from some recombinations that happened in the bulk, the experimental result can be more close to the simulated theoretical one. Compared with the reference sample, the BSF layer enhanced the *V*_oc_, *J*_sc_, and PCE by 4.5 mV, 2.1 mA/cm^2^, and 1.40 %, respectively. In theory, BSF can also improve the contact properties of moderately doped c-Si and mental electrode, resulting in the improvement of both FF and series resistance (*R*_s_). But in our experiment, we used the InGa alloy which has better contact properties as the back electrode instead of aluminum or other ordinary metals. So for the reference cells, the InGa alloy overcame the contact problem for the moderately doped substrate, resulting in a higher FF. As a result, the improvement effect of BSF on the FF and *R*_s_ parameters was not obvious as it should be when comparing the reference and BSF cells because the effect was masked by the excellent contact properties of the InGa alloy. The average *J*_sc_ of the devices combined with BSF and MoO_3_ AR coating is 28.7 mA/cm^2^, demonstrating an increase by 13.4 and 23.7 % in comparison with the reference and BSF devices, respectively. Compared to the simulated *J*_ph_ of 37.00 mA/cm^2^, the experimented *J*_sc_ of 28.7 mA/cm^2^ was lower than expected. This can be largely attributed to the recombination that occurred on the surface or in the bulk, which reduced the carrier density and thus caused the decrease of short-circuit current density.

**Table 1:** Performance indicators of Si/PEDOT:PSS heterojunction solar cells

	*V*_oc_ (mV)	*J*_sc_ (mA/cm^2^)	FF (%)	PCE (%)	*R*_s_ (Ω cm^2^)
Reference	572.6 ± 1.2	23.2 ± 0.1	69.3 ± 0.5	9.23 ± 0.20	5.5 ± 0.7
BSF	598.1 ± 0.4	25.3 ± 0.1	70.2 ± 0.6	10.63 ± 0.18	4.6 ± 0.9
BSF and MoO_3_	599.8 ± 0.8	28.7 ± 0.2	69.2 ± 0.8	11.90 ± 0.11	4.3 ± 1.0

Yiling Sun et al.

The EQE of each sample cell was measured, as shown in Fig. 3b. As expected, the cells with the MoO_3_ AR layer and BSF design displayed a higher EQE value in the visible and near infrared regions in comparison with the reference and BSF cells (which is consistent with the increase of *J*_sc_), benefiting from increased intensity of the incident light to the *p-n* junction. In the ultraviolet band, the unexpected lower EQE value was caused by parasitic absorption of MoO_3_ material. Unlike PEDOT:PSS, MoO_3_ displayed a high-extinction coefficient and parasitic absorption in this ultraviolet wavelength region (Figs. 1b and 2c).

**Fig. 4:**
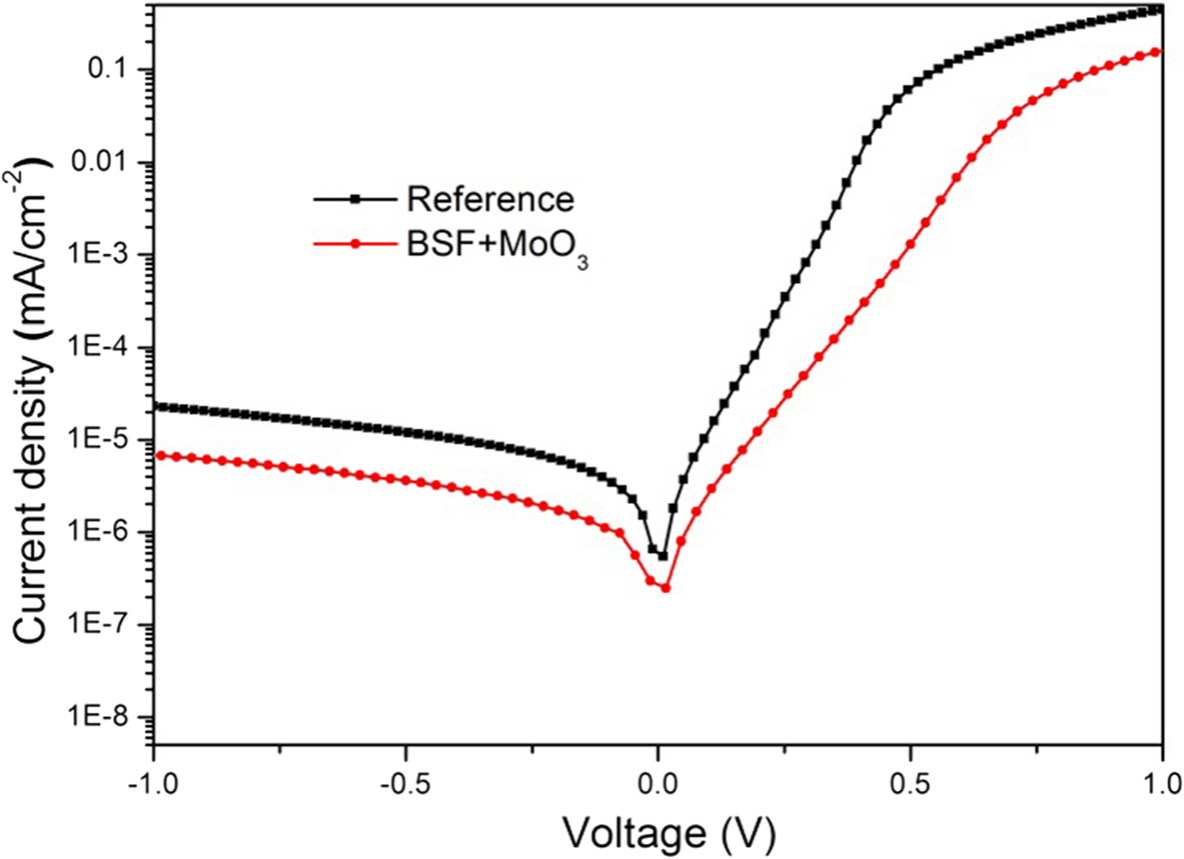
Dark *J-V* curve of Si/PEDOT:PSS devices with or without BSF layer

In order to further study the performance of the cells, the dark current density versus voltage characteristic of the hybrid solar cells with and without a BSF layer was measured, and the results were plotted (Fig. 4). It was observed that the current density (*J*_s_) was suppressed significantly after the BSF layer was inserted between Si and InGa. In order to extract the parameters of heterojunction as well as a diode, the dark *J-V* curves were simulated according to the thermionic emission model as follows:

1 $$ \mathrm{J}={\mathrm{J}}_{\mathrm{s}}\left[ \exp \left(\mathrm{eV}/\mathrm{n}\mathrm{k}\mathrm{T}\right)-1\right] $$

2 $$ {\mathrm{J}}_{\mathrm{s}}={\mathrm{A}}^{*}{\mathrm{A}\mathrm{T}}^2 \exp \left(-{\varPhi}_{\mathrm{bi}}/\mathrm{K}\mathrm{T}\right) $$

where *V* is the applied voltage, *T* is the absolute temperature (298 K), *n* is ideality factor, *k* is the Boltzmann constant (1.38 × 1023 m^2^ kgs^−2^ K^−1^), *q* is the electronic charge (1.6 × 10^−19^C), *A* is the contact area, A* is the effective Richardson constant (about 252 A cm^−2^ K^−2^ for *n*-type Si), and *Φ*_bi_ is barrier height of Schottky diode. By combining the thermionic emission model and the curve we tested, the *n*, *J*_*s*_, and *Φ*_bi_ of the heterojunction solar cells were extracted (see Table 2). The device with BSF layers exhibited a *J*_s_ value of 1.54 × 10^−7^ A/cm^2^, approximately a half of that of the reference device (which was 2.88 × 10^−7^ A/cm^2^). Here, the ideality factor *n* in this Schottky diode is linked to the quality of the *p*-*n* junction which is influenced by the recombination velocity on the surface of Si. The smaller *n* value of the BSF-inserted device (2.15) implied a suppressed recombination rate. Moreover, the improved *J*_0_ and *n* parameters are also a compelling evidence that the charge carriers were collected by the electrode with higher efficiency thanks to the improved contact ability of the BSF layer. In contrast, the value of *Φ*_bi_ in the Si/PEDOT:PSS junctions increased with the addition of the BSF layer, yielding a value of 0.821 eV for the BSF-inserted sample, an increase of 0.016 eV from the value of 0.805 eV of the reference cell. The increase of *Φ*_bi_ improved *J*_sc_ and *V*_oc_ of the Si/PEDOT:PSS heterojunction solar cells, achieved by reinforcing the internal electric field and charge separation force.

**Table 2:** Diode ideality factor (*n*), reverse saturation current density (*J*_*s*_), and Schottky barrier height (*Φ*_bi_) values of Si/PEDOT:PSS heterojunction solar cells

	*J*_*s*_ (A/cm^2^)	*n*	Φ_bi_ (eV)
Reference	2.88 × 10^−7^	2.45	0.805
BSF and MoO_3_	1.54 × 10^−7^	2.15	0.821

Yiling Sun et al.

## Conclusions

We have applied a simulation model to analyze the photocurrent of Si/PEDOT:PSS heterojunction solar cells. The optical properties we simulated matched well with the experiment results. By coating a MoO_3_ AR layer with a thickness of 34 nm, the performance of BSF-involved Si/PEDOT:PSS hybrid solar cells can be significantly improved. Solar cells with a higher PCE of 11.90 % was eventually achieved through enhancement of short-circuit current density.

## Abbreviations

AR, antireflection; ARCs: antireflection coatings; BSF, back surface field; *J*_sc_, short-circuit current density; c-Si, crystalline silicon; EQE, external quantum efficiency; FF, fill factor; *J*_loss_, loss current density; *J*_ph_, photocurrent density; *J*_tot_, total current density; *J-V*, current density-voltage; *k*, extinction coefficient; MoO_3_, molybdenum oxide; *N*, refractive index; PCE, power conversion efficiency; PEDOT:PSS, poly(3,4-ethylenedioxythiophene):poly(styrenesulfonate); *P*_loss_, loss percentage of total current density percentage; total loss percentage; *R*, reflection loss; *R*_s_, series resistance
